# Extracellular Vesicles from Different Pneumococcal Serotypes Are Internalized by Macrophages and Induce Host Immune Responses

**DOI:** 10.3390/pathogens10121530

**Published:** 2021-11-23

**Authors:** Alfonso Olaya-Abril, Rafael Prados-Rosales, José A. González-Reyes, Arturo Casadevall, Liise-anne Pirofski, Manuel J. Rodríguez-Ortega

**Affiliations:** 1 Departamento de Bioquímica y Biología Molecular, Campus de Excelencia Internacional CeiA3, Universidad de Córdoba, 14071 Córdoba, Spain; b22olaba@uco.es; 2 Department of Preventive Medicine and Public Health and Microbiology, Autonoma University of Madrid, 28029 Madrid, Spain; rafael.prados@uam.es; 3 Departamento de Biología Celular, Fisiología e Inmunología, Campus de Excelencia Internacional CeiA3, Universidad de Córdoba, 14071 Córdoba, Spain; bc1gorej@uco.es; 4 Department of Molecular Microbiology and Immunology, Johns Hopkins Bloomberg School of Public Health, Baltimore, MD 21205, USA; acasade1@jhu.edu; 5 Montefiore Medical Center, Department of Medicine and Microbiology and Immunology, Albert Einstein College of Medicine, Bronx, New York, NY 10467, USA; l.pirofski@einsteinmed.org

**Keywords:** host-pathogen interaction, *Streptococcus pneumoniae*, membrane vesicles, immune response

## Abstract

Bacterial extracellular vesicles are membranous ultrastructures released from the cell surface. They play important roles in the interaction between the host and the bacteria. In this work, we show how extracellular vesicles produced by four different serotypes of the important human pathogen, *Streptococcus pneumoniae*, are internalized by murine J774A.1 macrophages via fusion with the membrane of the host cells. We also evaluated the capacity of pneumococcal extracellular vesicles to elicit an immune response by macrophages. Macrophages treated with the vesicles underwent a serotype-dependent transient loss of viability, which was further reverted. The vesicles induced the production of proinflammatory cytokines, which was higher for serotype 1 and serotype 8-derived vesicles. These results demonstrate the biological activity of extracellular vesicles of clinically important pneumococcal serotypes.

## 1. Introduction

Pathogenic microorganisms have developed numerous strategies to survive within their hosts, defending from the immune system and evading its action. One of these mechanisms is the release of membrane-derived extracellular vesicles (EV) [1]. Traditionally, it was known that these structures are produced by Gram-negative bacteria, but recent evidence has revealed that they are also produced by Gram-positive [2]. EVs of disease-causing bacteria are important factors in host–pathogen interplay, as they can increase the pathogenicity of microbes by transferring virulence factors into host cells and exert cytotoxic effects [1,3]. In their cargo, EVs transport not only proteins, but also other molecules such as metabolites and nucleic acids, which can also play important roles in microbe-microbe interaction, biofilm formation or acquisition of resistance to antibiotics [4,5]. However, they can also have a beneficial effect by triggering an immune response in the host that induces cytokines to fight against infections caused by the bacteria that release them [6].

*Streptococcus pneumoniae*, also known as the pneumococcus, is a Gram-positive bacterium that inhabits the upper respiratory tract of humans. Under certain conditions, it has the capacity to cause local (e.g., otitis media, pneumonia) or invasive disease (e.g., bacteremia, meningitis) [7,8]. As with other pathogens, pneumococcal EVs may play important functions in the interaction with the host. We demonstrated for the first time that both encapsulated and non-encapsulated strains of this bacterium species produce EVs [9]. We also showed that the EVs had a biochemical composition different from the plasma membrane from which they derive, as they are more enriched in lipoproteins and short-chain fatty acids. In this work, we show that EVs from four different pneumococcal serotypes, including three encapsulated and clinically relevant, are internalized by murine J774A.1 macrophages after fusion with their membrane. Macrophages treated with the EVs did not lose viability in a dose-dependent manner, but did in a serotype-dependent way, which was further reverted. We also measured the production of a panel of cytokines by such macrophages in response to different doses of EVs. The vesicles induced the production of proinflammatory cytokines, which was higher for serotype 1 and serotype 8-derived vesicles. These results demonstrate that EVs from clinically relevant pneumococcal serotypes are also able to induce an immune response, as previously described for reference strains.

## 2. Results

### 2.1. Release of Pneumococcal EVs Cargo into Host Cells

**Figure 1:**
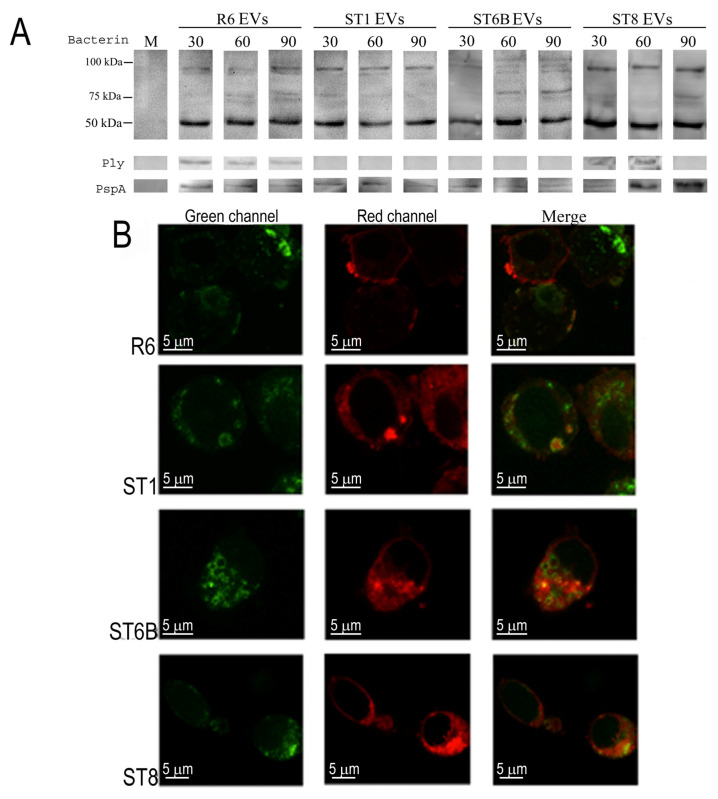
Fusion of pneumococcal extracellular vesicles (EVs) with the plasma membrane of cultured macrophages. (**A**) Transfer of EV content to macrophages analyzed by Western blotting. Total extracts of cultured macrophage cells were hybridized with anti-bacterin serum and sera raised against Ply and PspA. As a control (lane M), we used macrophages at time 0. (**B**) Macrophage cell membranes were labeled with the lipid rafts-marker CtxB conjugated with FITC (green fluorescence, left column), and EVs were labeled with rhodamine-R18 (red fluorescence). EVs (50 μg/mL) fused to macrophage membranes after 30 min incubation (second column). Merging of both fluorescence emission lights is represented in the right column.

We investigated whether EVs from the reference *S. pneumoniae* R6 non-encapsulated strain and three other encapsulated strains, belonging to clinically relevant serotypes, transferred their cargo to infected cells and the mechanism by which this occurred. We incubated murine J774A.1 macrophages with EVs and assessed the presence of pneumococcal proteins within cells at different times by immunoblot of macrophage cell lysates using an anti-bacterin serum (i.e., a serum against killed bacteria). These experiments revealed that the EV content was transferred to the macrophages as early as 30 min after incubation (Figure 1A). In contrast, no bacterial proteins were detected in controls (non EV-treated macrophages). The same results were observed when we used antibodies to Ply and PspA, as we previously demonstrated that these proteins are contained in the EV cargo [9]. However, for Ply, the transfer of the protein to the macrophages was not so clearly observed for EVs of the strains ST1 and ST6B.

We studied whether this transfer occurred via fusion of EVs with the macrophage plasma membrane. EVs were labeled with rhodamine-R18, a fluorescent dye that emits light after dequenching, when its concentration diminishes. The rationale for this experiment was to add a high concentration of the dye to the EVs, such that it would be quenched and no emission could be recorded. Light emission would only occur after fusion with another membrane because of rhodamine-R18 dequenching by dilution. Confocal microscopy analysis of cultured macrophages revealed a clear increase in red fluorescence after a 30-min incubation with rhodamine-labeled EVs that was not observed in non EV-treated macrophages (Figure 1B).

**Figure 2:**
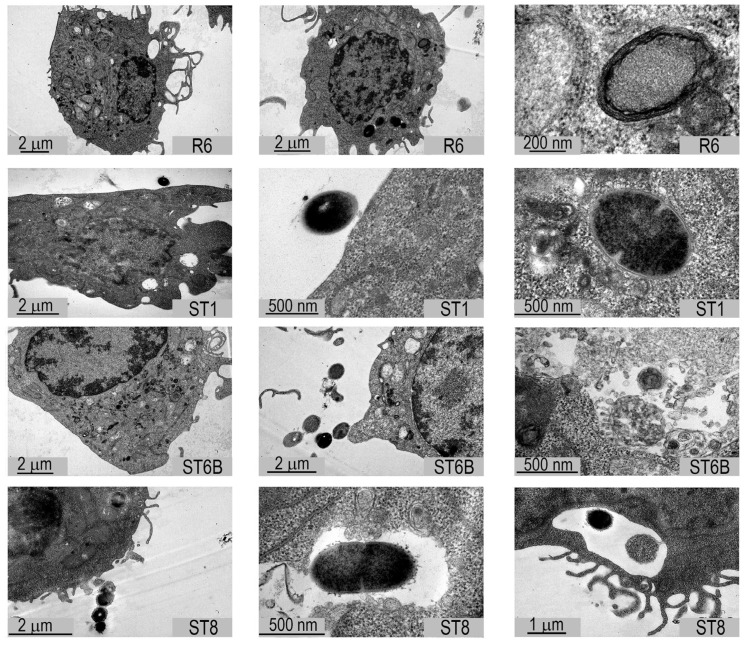
Transmission electron microscopy of macrophages infected with pneumococcus. Shown here are pictures of murine J774A.1 macrophages after 1 h of infection with R6, ST1, ST6B and ST8 pneumococcal strains (three examples per each strain, each one in a row).

We also tested the ability of bacteria to release EVs within the macrophages after they had been ingested by phagocytosis, using transmission electron microscopy of the macrophages infected with the four pneumococcal strains. However, EV release could not be clearly observed (Figure 2).

### 2.2. Pneumococcal EVs Stimulation of Host Cells

**Figure 3:**
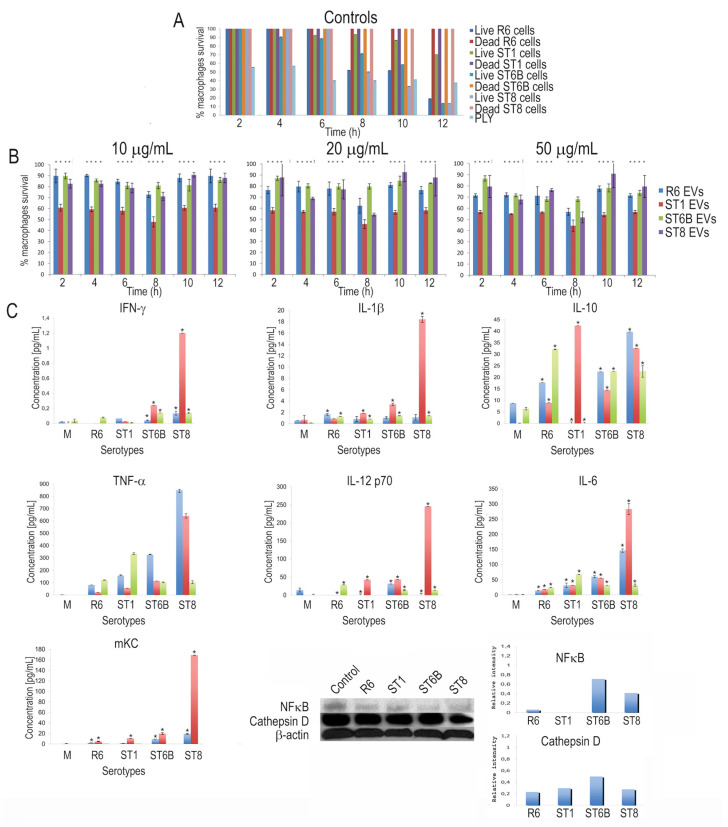
Cultured murine macrophage responses to membrane extracellular vesicle (EV) stimulation. (**A**) Survival MTT assay of cultured macrophages representing the controls of EV stimulation. As positive controls, macrophages were infected with the different pneumococcal strains or were treated with pneumolysin. As negative controls, macrophages were infected with killed bacteria. (**B**) Time-dependent survival MTT assay of macrophages stimulated with EVs from the different pneumococcal strains at three EV concentrations. (**C**) Cytokine production of macrophages in response to EV stimulation at a concentration of 20 μg/mL. Color bar codes are 6 h for blue, 9 h for red and 12 h for green. Amounts of cathepsin D and NF-κB were normalized with actin.

We assessed the ability of pneumococcal EVs to elicit a response in cultured cells. Murine J774A.1 macrophages were cultured with different concentrations of EVs (10, 20 and 50 μg/mL) and assayed for survival. First, we used the MTT cytotoxicity assay, which measures cell death, preferentially in an apoptotic way. As positive controls, we infected the macrophages with each strain (MOI = 10:1); as negative controls, we used the same bacteria after killing by treatment with isopropanol for 1 h (Figure 3A). As expected, live bacteria started to induce cell death after a 6 h incubation time, whereas killed bacteria did not. When EVs were added to the cultured macrophages, cell death occurred, but it was not, in general, in a dose-dependent manner at the doses tested, except for the R6 strain at shorter times (Figure 3B). For all doses and strains, a transitory cell death effect was observed, with the highest death rates occurring 8 h after EV stimulation. In all cases, cytotoxic effects disappeared with time as survival recovered to initial rates. The highest cell death rates corresponded to EVs from serotype 1.

Finally, we evaluated the capacity of EVs to produce a pro-inflammatory response in macrophages by measuring cytokine production after stimulation with an EV concentration of 20 μg/mL (Figure 3C). We used a multiplex assay, including six representative cytokines and one chemokine (IFN-γ, IL-10, IL-12 p70, IL-1β, IL-6, TNF-α and mKC). For most EV doses, the peaks of production occurred 9 h after stimulation. EVs from serotype 8 caused a stronger induction effect than the other serotypes. EVs also induced the production of cathepsin D, a well-known marker of apoptosis, thus supporting cytotoxicity data obtained with the MTT assay. Finally, we also measured the levels of NF-κB as a mediator of the cellular response to infection and other types of stress. NF-κB was also induced in the macrophages by EV stimulation. Together, these results indicate that pneumococcal EVs are biologically active and induce an immune response in cultured cells.

## 3. Discussion

EVs are membranous structures released by many microorganisms with a wide range of biological functions, including immunomodulatory effects on/evasion from the host immune system, as well as pathogenicity weapons [10,11]. In a previous study, we demonstrated that pneumococcus produces EVs that are biochemically different from the plasma membrane and that their cargo includes many surface proteins and virulence factors, including pneumolysin [9]. This has also been reported very recently for the TIGR4 strain [12]. Moreover, we showed that these EVs are highly immunoreactive and protect mice against infection. Recently, we reported that the amount of EVs produced by the four pneumococcal strains used in this work vary according to the growth medium used, and EVs increase when grown in a medium that has been previously conditioned by macrophages [13]. In this study, we delved deeper into the nature of pneumococcal EVs to gain more knowledge about their biological functions when interacting with host cells. Codemo et al. reported that EVs from TIGR4 are internalized by A549 and monocyte-derived dendritic cells [12]. More recently, other works showed very similar results using EVs derived from the R6 strain [14,15]. Here, we show that EVs from both the non-capsulated R6 and clinically-relevant encapsulated strains transfer their content into macrophages via membrane fusion, a mechanism described for EV entry into host cells [16]. For both Gram-negative and Gram-positive bacteria, this fusion is cholesterol-dependent and mediated by lipid rafts [17,18,19]. We tried to test this with pneumococcal EVs and J774A.1 macrophages using the cholesterol-blocking agent Filipin III, but positive results were not obtained (results not shown). We could not definitively demonstrate that the fusion of EVs to the macrophage cell membrane occurred through lipid rafts, as clear overlaps of rhodamine-R18 and FITC-CtxB were not observed. Nonetheless, our confocal microscopy data clearly showed that EVs fused to host cell membranes, regardless of the mechanism by which this occurs. The EV content was transferred to macrophages as soon as 30 min after contact, as revealed by Western blot analyses. To understand whether EVs could be produced in vivo within host cells, we infected macrophages with ST8. However, we could not observe EV release from bacteria in phagolysosomes, with the caveat that this could be a false-negative result from insufficient sampling of EM images.

The proposed biological functions of bacterial EVs include that they can subvert the host immune system, diverting its efforts towards such vesicles instead of bacterial cells. At the same time, EVs could function as offensive weapons that microbes may use to damage the host immune system from a distance [20,21]. Here, we report that stimulation of cultured macrophages with pneumococcal EVs has a transient effect on survival, causing a slight decrease in viability at short times that was followed by a reversion of the effect. Such an effect was not dose-dependent except for R6 at shorter times. We do not know whether this was due to the strong effect of EVs at the doses tested, i.e., lower doses might have caused a more pronounced dose-dependent response. On the other hand, the decrease in viability was more pronounced for ST1-derived EVs, and to a lesser extent, for EVs from ST8. Most ST8 strains possess a non-hemolytic Ply version but are able to evade host defense [22,23]. ST1 pathogenicity and cell cytotoxicity seem to be related to higher pneumolysin release compared to other serotypes [24]. Prevalent African hypervirulent serotype 1 has two pneumolysin variants, one linked to the cell wall, and the second one located in the cytoplasm [25]. This is in agreement with our results, in which we described previously that pneumolysin in our ST1 strain was found in total bacterial cell extracts and in secreted fractions, but not in the EVs [9]. This is in concordance with the results presented here, showing that pneumolysin was absent in macrophage extracts treated with ST1 EVs. Therefore, the higher decrease in cell viability caused by ST1-derived EVs is presumably not necessarily due to pneumolysin cargo. 

The cell death described here might take place preferentially by apoptosis, as macrophages were sensitive to the MTT assay and because of the induction of cathepsin D. In addition, no necrotic cell death was observed with the LDH cytotoxicity assay (data not shown). These results suggest that host cell death is more likely to occur by apoptosis than necrosis, although we cannot rule out that both occur. Previous reports describe the cytotoxic effect of bacterial EVs on host cells, both in Gram-positives [18,19] at long time exposures (24 h) of epithelial cells to *S. aureus* EVs, and Gram-negatives, at shorter exposures (8 h) of epithelial cells to *P. aeruginosa* OMVs [17]. We also show that pneumococcal EVs promote a proinflammatory response in macrophages as measured by cytokine production, consistent with previous reports on *S. aureus* [26], *Mycobacterium* [27] and *S. pyogenes* [28,29], as well as in the recent published works for pneumococcus [12,14,15].

In conclusion, our work shows that pneumococcal EVs from different serotypes, including clinically-relevant encapsulated ones, modulate the host immune system, inducing a proinflammatory response and transitory death of macrophages, which is mediated by their fusion with the plasma membrane of host cells and transfer of their cargo. The intensity of the immune response depends on the pneumococcal serotype producing such vesicles, although such a response does not vary significantly in qualitative terms. A better comprehension of EV-mediated bacteria–host interaction will contribute to more efficiently fighting against bacterial infections.

## 4. Materials and Methods

### 4.1. Cell Lines, Bacterial Strains and Growth 

J774A.1 macrophages were cultured at 37 °C in 5% CO_2_ atmosphere in air, in Dulbecco’s modified Eagle’s medium (DMEM) supplemented with 10% fetal bovine serum, 10% NCTC and 1% non-essential amino acids. Four *Streptococcus pneumoniae* strains (R6, serotype 2; ST1, serotype 1; ST6B, serotype 6B; ST8, serotype 8) were grown at 37 °C in 5% CO_2_ atmosphere in air in Todd-Hewitt broth (THB) until they reached their respective mid-exponential phase.

### 4.2. Pneumococcal EVs Production and Quantification 

EVs were isolated as described [9] by using a series of Optiprep gradient layers with concentrations ranging from 35%–5% (*w/v*). Briefly, cells at different ODs were pelleted from 1-L cultures, and the supernatants were filtered through 0.22 μm pore size filters (Millipore). The supernatants were then centrifuged at 100,000× *g* for 1.5 h at 4 °C to sediment the vesicular fraction. The pellets were mixed with 2 mL of Optiprep solution (Sigma-Aldrich, St. Louis, MO, USA), yielding 35% (*w/v*) Optiprep final concentration. The crude vesicle sample was then overlayed with a series of Optiprep gradient layers with concentrations ranging from 35% to 5% (*w/v*). The gradients were centrifuged (100,000× *g* for 16 h at 4 °C), and 1 mL fractions were removed from the top. The fractions were then centrifuged at 100,000× *g* for 1 h at 4 °C and recovered. Finally, vesicles were air-dried, weighed and suspended in PBS.

### 4.3. Capsule Quantification 

The amount of capsule was determined using the Stains-all assay (Sigma-Aldrich) for detecting acidic polysaccharides, as described previously [30]. The bacteria were cultured to late-exponential phase, then 5 mL were centrifuged for 10 min at 5000× *g* and 4 °C, washed with PBS and resuspended in 0.5 mL 0.85% NaCl. Ten μl were removed to make dilutions in PBS for plating out to quantify the number of bacteria. For the remaining bacterial suspension, 2 mL of a solution containing 20 mg of 1-ethyl-2(3-(1-ethylnaphthho-(1,2-d)thiazolin-2-ylidene)-2methylpropenyl)naphthho-(1,2d)thiazoliumbromide (Stains-all) and 60 mL of glacial acetic acid in 100 mL 50% formamide were added, and the OD_640_-determined 0.5 mL of 0.85% NaCl with 2 mL Stains-all solution was used as a blank. 

### 4.4. Western Blotting 

To detect the presence of pneumococcal proteins in J774A.1 macrophages after stimulation with EVs, 400 μg of macrophage total extract were loaded in a 12% SDS-PAGE in the case of Ply and PspA, and 200 μg in the case of bacterin (serum from mice infected with isopropanol-innactivated pneumococcal strain R6), and then transferred to PVDF membranes. Anti-Ply (Abcam, Cambridge, UK) and anti-PspA (Thermo Scientific, San Jose, CA, USA) antibodies were used at 1:1000. For NFƙB and Cathepsin D analysis, 30 μg of macrophage lysates were separated by SDS-PAGE, transferred to PVDF membranes and incubated with 1:1000 rabbit NFƙB–antibody (Invitrogen, Waltham, MS, USA), 1:200 goat polyclonal IgG Cathepsin D antibody (Santa Cruz Biotechnology, Dallas, TX, USA) and 1:5000 β-actin HRP-conjugated (Santa Cruz Biotechnology). Measurements were performed using ImageJ 1.40 software (N.I.H., Bethesda, MD, USA). 

### 4.5. Microscopy

For transmission electron microscopy, samples were fixed in 2.5% glutaraldehyde/2% paraformaldehyde in a 0.1 M sodium cacodylate buffer for 4 h and post-fixed in 1% osmium tetroxide for 1 h at 4 °C in the same buffer. After dehydration in an ascendant series of ethanol, the pieces were transferred to propylene oxide and sequentially infiltrated in Embed 812 resin. After staining in aqueous 4% uranyl acetate and ready-to-use lead citrate (EMS, Hatfield, PA, USA), the sections were observed in a Jeol JEM 1400 electron microscope. Confocal microscopy was carried out using a NIKON D-Eclipse C1 Confocal Laser with a NIKON Eclipse 90i Microscope. Fluorescence was recorded at 488 nm (green; FITC) and 543 nm (red; rhodamine isothiocyanate B-R18). Fluorescence microscopy was carried out as described [31].

### 4.6. MTT Assay 

Growth of macrophages was assessed with the MTT (3-(4,5-dimethylthiazol-2-yl)-2,5-diphenyl tetrazolium bromide) assay. Briefly, 5 × 10^4^ cells were added into each well in a 96-well plate. After 24 h, samples (pneumococcus, EVs or Ply) at different concentrations were added. After stimulation (2, 4, 6, 8, 10 and 12 h), 175 µL of medium were removed, and MTT was added for 2 h. Then, 100 µL of dimethyl sulfoxide was added to wells, and absorbance at 570 nm was recorded in a microplate reader after over-night incubation. 

### 4.7. Cytokine Measurement 

Macrophage culture supernatants stimulated with 20 µg/mL EVs for 6, 9 and 12 h were assayed for a panel of mouse proinflammatory cityokines (IFN-γ, IL-10, IL-12p70, IL-1β, IL-6, IL-8, TNF-α) using the 7-Plex Ultra-Sensitive ELISA Kit (Meso Scale Discovery, Rockville, MD, USA), according to the manufacturer’s specifications.

### 4.8. Membrane Fusion Assay 

To assess membrane fusion using *S. pneumoniae* EVs and J774A.1 macrophage cells, we essentially followed procedures described earlier [19]. Briefly, EVs were labeled with 1 mg/mL rhodamine isothiocyanate B-R18 (Molecular Probes) for 1 h at room temperature. An unlabeled probe was removed by centrifugation at 100,000× *g* (60 min, 4 °C). After washing with PBS, labeled EVs were resuspended in 1 mL PBS. Subsequently, the host cell plasma membrane was labeled for 1 h prior to the incubation with EVs with 8 mg/mL FITC-conjugated cholera toxin B subunit (CtxB) (Sigma-Aldrich). Then, labeled EVs were added in a 1:4 dilution in the wells and incubated for 30 min at 37 °C. When applicable, 10 mg/mL Filipin III was added 30 min prior to the addition of EVs. After incubation with EVs, cell samples were analyzed by confocal microscopy as described above.

### 4.9. Statistics

Statistical analyses were performed using SPSS v 21.0.0.0. A Student’s *t*-test (2-tailed) was applied for experiments involving pairwise comparisons, and *p* < 0.05 was considered significant.

## Data Availability

No new data were created or analyzed in this study. Data sharing is not applicable to this article.
